# Adherence to hemodialysis and medical regimens among patients with end-stage renal disease during COVID-19 pandemic: a cross-sectional study

**DOI:** 10.1186/s12882-022-02756-0

**Published:** 2022-04-09

**Authors:** Basma Osman Sultan, Ahmed Mahmoud Fouad, Heba M. Zaki

**Affiliations:** 1 Department of Internal Medicine, Nephrology Unit, Faculty of Medicine, Suez Canal University, Kilo 4.5, Ring Road, P.O: 41522, Ismailia, Egypt; 2 Department of Public Health, Occupational and Environmental Medicine, Faculty of Medicine, Suez Canal University, Ismailia, Egypt

**Keywords:** Adherence, Fear of COVID-19, End stage renal disease, Hemodialysis, Egypt

## Abstract

**Background:**

Adherence of patients with End-Stage Renal Disease (ESRD) to Hemodialysis (HD), prescribed medications, diet and fluid restrictions is essential to get the desirable outcome and prevent complications. During COVID-19 pandemic, ESRD patients became more concerned with attending the HD sessions and following the protective measures because of the potential for increased susceptibility to COVID-19. The aim of this study was to evaluate the impact of the pandemic on patients' adherence to HD and medical regimens.

**Methods:**

Two hundred five ESRD patients on HD were interviewed with the ESRD Adherence Questionnaire (ESRD-AQ) and the Fear-of-COVID-19 Scale (FCV-19S). Clinical and laboratory correlates of adherence were retrieved from patients' records.

**Results:**

Self-reported adherence to HD showed that 19.5% were not adherent to HD during the pandemic compared to 11.7% before the pandemic (*p* < 0.001), with a significant agreement with the actual attendance of HD sessions (Kappa = 0.733, *p* < 0.001). Twenty-five patients (12.2%) had a history of COVID-19. The FCV-19S had a mean score of 18.8 and showed significant positive correlations with the pre-dialysis phosphorus and potassium. Multivariate analysis showed that the main predictors of non-adherence were the history of COVID-19, understanding and perception scores, and the Fear-of-COVID score.

**Conclusions:**

The COVID-19 pandemic adversely affected the adherence of ESRD patients to HD and medical regimen. Strategies to mitigate patients' fears of COVID-19 and improve their understanding and perceptions of adherence to HD and medical regimen should be adopted in HD centers during the pandemic.

**Supplementary Information:**

The online version contains supplementary material available at 10.1186/s12882-022-02756-0.

## Introduction

Adherence was defined as "the extent to which a person's attitude matches with the agreed recommendations of a healthcare giver in terms of taking medications, following a recommended diet regimen and/or carrying out lifestyle changes" [1]. Adherence to medications is a major challenge in patients with chronic diseases since non-adherence to was usually associated with disease deterioration and increased hospital admissions [2].

End-Stage Renal Disease (ESRD) is a major public health problem that has been associated with a growing burden on healthcare systems and the economy worldwide [3]. Non-adherence to treatment is a common behavior among patients with ESRD, which has been associated with unfavorable consequences, such as bone demineralization, pulmonary edema, metabolic disorders, and increased mortality [4]. In Dialysis Outcomes and Practice Patterns Study (DOPPS), a large prospective observational study for the outcomes of hemodialysis practice, non-adherence to hemodialysis (HD), dietary and fluid restrictions, and medical treatment were significantly associated with increased hospital admissions and mortality [5].

Since December 2019, the world is struggling with the Corona-virus disease (COVID-19) pandemic and its adverse effects on the healthcare systems and economies [6]. Patients with chronic diseases such as diabetes, hypertension, cardiovascular diseases were at greater risk for COVID-19 related morbidity and mortality. During the pandemic, patients with ESRD were of particular concerns. They have increased rates of comorbidities, hospitalization and deaths, which adds up to the burden of COVID-19. Patients on HD are at increased risk for SARS-CoV-2 infection and the worst outcomes of COVID-19, because they are usually elderly and have a high prevalence of diabetes mellitus and hypertension [7, 8]. Moreover, patients on HD have to leave their home many times per week, regardless of stay-at-home orders, whereas they cannot avoid exposure to others on mass transportation, shared transportation rides, or in waiting rooms of the dialysis center [9, 10].

As the pandemic continued, more information become available on the psychosocial impact of the pandemic on general population and more deleterious on patients with chronic diseases [11]. People with chronic diseases, and particularly ESRD, had more fears and worries about the COVID-19 and were struggling with poor psychological well-being and fewer resources than healthy people [12, 13].

Therefore, the purpose of this study was to evaluate the impact of the COVID-19 pandemic and associated patients' fears on adherence to HD and medical treatment among ESRD patients.

### Patients and methods

This cross-sectional study was conducted at the dialysis centers in Ismailia governorate, Egypt, during the last quarter of 2020. A sample size of 205 patients was calculated using Epi-InfoTM Software version 7.2.4.0 (Centers for Disease Control and Prevention, Atlanta, GA, USA) which was enough to detect an expected percentage of at least 14% non-adherence rate among ESRD patients, at 95% level of confidence, 5% absolute precision, and 10% dropout.

Inclusion criteria: adult patients (≥ 18 years) with ESRD who were on maintenance HD of at least twice weekly for a minimum of one-year duration.

Exclusion criteria: patients who were not conscious or not able to give their written consent, and patients with incomplete medical records.

Ethical approval was obtained from the ethical committee at the faculty of medicine, Suez Canal University, Egypt. All patients gave their written consent prior to their participation in this study. All selected patients were interviewed using the End-Stage Renal Disease Adherence Questionnaire (ESRD-AQ) [14] which comprised 46 items involving five sections: general patient information, attendance of HD session, adherence to medications, fluid restriction, and diet recommendations. Further details of ESRD-AQ's items, subscales, and scoring were described by Kim et al. [14]. ESRD-AQ is a valid instrument for assessment of adherence among HD patients. The Arabic–translated version of the ESRD-AQ is available from Naalweh et al. [15]. A total score for adherence behavior of less than 700 was interpreted as poor or non-adherence, while a score of 700–999 indicated moderate adherence and 1000–1200 indicated a good adherence [15].

Fear-of-COVID-19 Scale (FCV-19S) is a recent scale for assessment of specific anxieties regarding the COVID-19 pandemic. The FCV-19S is a seven-item questionnaire rated on a five-point Likert scale, where "strongly disagree" equals 1, "disagree" equals 2, "neutral" equals 3, "agree" equals 4, and "strongly agree" equals 4. The total score is calculated by summing up all items scores, and ranges from 7 to 35 [16]. The Arabic-translation of FCV-19S was validated by Alyami et al. [17].

Clinical and laboratory correlates of adherence were retrieved from the most recent patients' records. For example, the pre-dialytic serum potassium and phosphate levels, and the inter-dialytic body weight (IDW)—calculated as the difference between the patients' weight measured at the onset of a dialysis treatment and their weight at the end of the previous dialysis session—were used as indicators for adherence to diet, medications, and fluid restriction regimen, respectively. Further laboratory records were retrieved for hemoglobin level, PTH level, and serum calcium, ferritin and albumin levels.

### Statistical analysis

All data manipulation and analyses were performed with SPSS® Statistics version 25 (IBM Corporation, Armonk, NY, USA). Data normality was tested with the Smirnov–Kolmogorov test. Continuous variables were summarized as the mean (Standard Deviation), while categorical variables were described as frequencies and percentages (%). Associations between categorical variables were tested for statistical significance using chi-square or Fisher's exact tests (if > 20% of expected values were less than 5), while the correlations between continuous variables were performed with Spearman's correlations. Wilcoxon Signed-Ranks Test was used to test for difference in adherence scores before and during the COVID-19 pandemic. McNemar test was used to test significance between the pre- and during the pandemic adherence levels. Multivariable logistic regression model was used to assess the association between non-adherence as an outcome and demographic and clinical variables. Variables with a *p*-value less than 0.20 on bivariate analyses were selected to enter the model. Removal of variables from the model was based on insignificant change in the model's R-square. A *p*-value of < 0.05 was considered statistically significant.

## Results

This study involved 205 ESRD patients with 64% males and 36.1% females. Patients' age ranged from 18 to 82 years, with a mean of 45.9 years. About two-thirds of patients were married and one-third did not have offspring. Half of the patients completed their high school, 27.3% had a university degree, 13.2% had basic education or less and 9.8% were illiterate. More than half the patients were not working or retired and about 10% were housewives. Over two-thirds of the patients were living at urban areas. About 70% of the patients had other chronic health conditions. The most frequent chronic comorbidities were hypertension, diabetes mellitus and cardiac diseases (57%, 20%, 20% respectively). Only 1.5% of patients were not insured (i.e. out-of-pocket spending). The most common causes for hemodialysis in our patients were CKD of unknown etiology and hypertension (38% and 36% respectively). About 56% of the patients were on hemodialysis for five years or more. Only one patient had had a history of chronic peritoneal dialysis who was shifted to HD one-year prior to starting this study. Likewise, 18 (8.8%) patients had had a history of kidney transplantation, but they developed chronic rejection and were maintained on HD before the study. Forty-three percent of the patients reported private transportation as the mean transportation method to the hemodialysis center. Sixty percent were not accompanied by anyone on their way to the center. Twenty-five patients had a past history of COVID-19, either clinically suspected (12 patients, 5.9%) or PCR-confirmed diagnosis of SARS-CoV-2 infection (13 patients, 6.3%) (Table 1). About two-thirds of patients were adherent to the social distancing and home-isolation measures, while most patients were adherent to other protective measures such as mask and gloves (85.4%); or disinfection and hand washing (91.7%).

**Table 1:** Distribution of the studied patients by their sociodemographic and health-related characteristics (*N* = 205)

Characteristics	No. (%)
**Age**, mean (SD), range	45.9 (18.6), 18—82
**Gender**	
Male	131 (63.9%)
Female	74 (36.1%)
**Marital status**	
Single	56 (27.3%)
Married	134 (65.4%)
Divorced or Widowed	15 (7.3%)
**Number of offspring**	
None	68 (33.2%)
1 – 2	52 (25.4%)
More than 2	85 (41.5%)
**Education level**	
Illiterate	20 (9.8%)
Basic education or Less	27 (13.2%)
Secondary (High school)	102 (49.8%)
University	56 (27.3%)
**Work**	
Retired/ not working	112 (54.6%)
Housewives	20 (9.8%)
Employed**/** own business	73 (35.6%)
**Residence**	
Urban	142 (69.3%)
Rural	63 (30.7%)
**Number of comorbidities**	
None	63 (30.7%)
Single	77 (37.6%)
Multiple	65 (31.7%)
**Health Insurance Plan**	
Social health insurance	71 (34.6%)
Private health insurance	61 (29.8%)
State expense	70 (34.1%)
Out-of-Pocket	3 (1.5%)
**Duration of HD (years, *mean (SD), range***	6 (5.4), 0.2 – 25
Less than 5	94 (45.9%)
5 – 10	55 (26.8%)
Longer than 10	56 (27.3%)
**How he/she reached the center?**	
Public transportation	114 (55.6%)
Private transportation	88 (42.9%)
Ambulance	3 (1.5%)
**Who accompanied the patient to HD center?**	
None	121 (59.0%)
Family member	70 (34.1%)
Friend/ others	14 (6.8%)
**History of COVID-19**	25 (12.2%)

*PCK* Polycystic Kidney, *HD* Hemodialysis, *SD* Standard deviation

Adherence to medications and hemodialysis was self-reported by the ESRD-AQ. Subdomains and items scores of the ESRD-AQ were summarized in the Supplementary table 1 (S1). There was a significant agreement between the self-reported attendance and the actual attendance (retrieved from patients' medical records) of the hemodialysis sessions (Kappa = 0.733, *p* < 0.001). Self-reported attendance was less than actual attendance in 28 patients (13.7%). Forty patients (19.5%) were not adherent during the COVID-19 pandemic, compared to 24 (11.7%) before the pandemic, with a statistically significant difference. Sixteen (7.8%) patients who were adherent before the pandemic turned to non-adherent during the pandemic, while 15 (7.3%) patients with pre-pandemic good adherence turned to moderate adherence during the pandemic (Table 2).

**Table 2:** Distribution of the studied patients by their attendance status pre- and during the pandemic (*N* = 205)

**Adherence to Dialysis**		**During COVID-19 Pandemic ***no. (row %)*			**Total** *no. (column%)*	***p*-value**
		**Poor**	**Moderate**	**Good**		
**Pre-COVID-19 pandemic**	**Poor**	24 (100.0%)	0	0	24 (11.7%)	0.000*
	**Moderate**	12 (12.4%)	85 (87.6%)	0	97 (47.3%)	
	**Good**	4 (4.8%)	15 (17.9%)	65 (77.4%)	84 (41.0%)	
	**Total**	40 (19.5%)	100 (48.8%)	65 (31.7%)	205 (100.0%)	

^*^Statistically significant at *p* < 0.05, McNemar-Browker Test

**Fig. 1:**
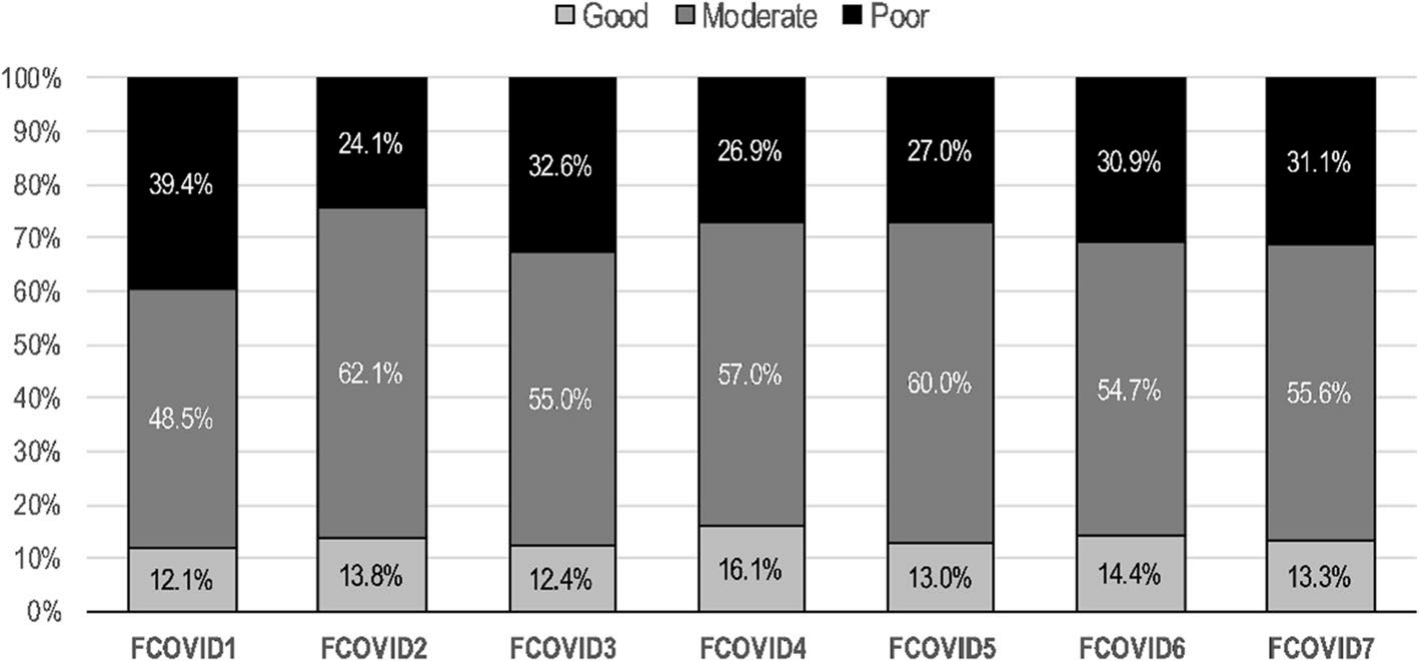
Distribution of patients with ERD who endorsed "agree/ strongly agree" of Fears-of-COVID-19 statements by their adherence behavior Level (good, moderate, and poor), (FCOVID: Fears-of-COVID item number)

Patients scored 7 to 35 on the Fears of COVID-19 scale, with a mean score of 18.8. Detailed patients' responses were summarized in the Supplementary table 2 (S2). Distribution of patients who agreed with Fears of COVID's statements by their adherence behavior was described in Fig. 1. The total adherence behavior score showed significant positive correlations with adherence perceptions and understanding, and the serum ferritin level, and a significant negative correlation with the FCV-19S. Likewise, adherence understanding score have a significant negative correlation with the FCV-19S and PTH levels. FCV-19S score showed significant positive correlations with pre-dialysis phosphorus and potassium, and a significant negative correlation with pre-dialysis PTH (Supplementary table S3).

In Table 3, there were no statistically significant differences between adherent and non-adherent patients regarding their demographic and clinical characteristics except for the history of COVID-19, fear-of-COVID score, and the adherence-related understanding and perception scores. Non-adherent patients showed significantly higher fears-of-COVID score and less understanding and perception scores than adherent patients. Likewise, the most recent laboratory investigations and intradialytic weight gain did not show significant differences between adherent and non-adherent patients except for the pre-dialysis potassium level. Non-adherent patients had significantly higher pre-dialysis potassium than adherent patients.

**Table 3:** Distribution of patients' characteristics by their adherence status medications and hemodialysis (*N* = 205)

Characteristics	n	Medical adherence during COVID-19 Pandemic *no. (row %)*		*p*-value
		**Adherent** (*n* = 165)	**Non-adherent** (*n* = 40)	
Age (years) mean (SD)	205	47.4 (18.6)	39.6 (17.7)	0.145
Gender				
Male	131	107 (81.7%)	24 (18.3%)	0.567
Female	74	58 (78.4%)	16 (21.6%)	
Marital status				
Single	56	40 (71.4%)	16 (28.6%)	0.123
Married	134	113 (84.3%)	21 (15.7%)	
Divorced or Widowed	15	12 (80.0%)	3 (20.0%)	
Number of offspring				
None	68	52 (76.5%)	16 (23.5%)	0.382
1 – 2	52	45 (86.5%)	7 (13.5%)	
More than 2	85	68 (80.0%)	17 (20.0%)	
Education				
Illiterate	20	14 (70.0%)	6 (30.0%)	0.606
Basic education or Less	27	22 (81.5%)	5 (18.5%)	
Secondary education	102	82 (80.4%)	20 (19.6%)	
College and above	56	47 (83.9%)	9 (16.1%)	
Employment				
Retired/ not working	112	90 (80.4%)	22 (19.6%)	0.771
Housewives	20	15 (75.0%)	5 (25.0%)	
Employed	73	60 (82.2%)	13 (17.8%)	
Residence				
Urban	142	115 (81.0%)	27 (19.0%)	0.787
Rural	63	50 (79.4%)	13 (20.6%)	
Number of Comorbid conditions				
None	63	46 (73.0%)	17 (27.0%)	0.195
Single	77	65 (84.4%)	12 (15.6%)	
Multiple	65	54 (83.1%)	11 (16.9%)	
Health Insurance Plan				
Social health insurance	71	59 (83.1%)	12 (16.9%)	0.788 ^f^
Private health insurance	61	49 (80.3%)	12 (19.7%)	
State expense	70	54 (77.1%)	16 (22.9%)	
Out-of-Pocket	3	3 (100.0%)	0 (0.0%)	
Duration of HD (years); mean (SD)	205	6.6 (5.5)	6.5 (4.8)	0.701
How he/she reached the center?				
Public transportation	117	96 (82.1%)	21 (17.9%)	0.515
Private transportation	88	69 (78.4%)	19 (21.6%)	
Who accompanied the patient to HD center?				
None	121	96 (79.3%)	25 (20.7%)	0.478
Family member	70	59 (84.3%)	11 (15.7%)	
Friend/ others	14	10 (71.4%)	4 (28.6%)	
History of COVID-19	25	14 (56.0%)	11 (44.0%)	0.002 *
Fears-of-COVID Scale, mean (SD)	205	18.0 (7.8)	23.6 (8.8)	0.000 *
Adherence-related perception score, mean (SD)	205	16.9 (1.9)	15.7 (2.5)	0.002 *
Adherence-related understanding score, mean (SD)	205	4.0 (0.1)	3.7 (0.7)	0.000 *
Most Recent laboratory investigations, mean (SD)				
Hemoglobin (g/dL)	205	10.0 (1.7)	9.5 (2.2)	0.108
Ferritin (μg/L)	205	501.2 (318.3)	484.1 (273.0)	0.265
Calcium (mg/dl)	205	8.6 (0.9)	8.2 (1.2)	0.069
PTH (ng/L)	205	464.1 (429.4)	389.0 (275.3)	0.299
Predialysis Phosphorus (mg/dl)	205	4.9 (1.3)	5.1 (1.4)	0.456
Predialysis Potassium (mg/dl)	205	5.0 (0.8)	5.1 (1.4)	0.021 *
Albumin (g/dl)	205	4.1 (0.5)	4.0 (0.5)	0.329
Intradialysis weight gain (IDW), kg, mean (SD)	205	2.7 (0.9)	3.1 (1.0)	0.070

*PTH* Parathyroid Hormone, *SD* Standard Deviation, *f.* fisher's exact test, *HD* hemodialysis

^*^Statistically significant at *p* < 0.05

Multivariate analysis showed that the main predictors of non-adherence to HD and medications were the history of COVID-19, understanding and perception scores, and the Fear-of-COVID score. Patients with a history of COVID-19 were 5.36 times more likely to be non-adherent compared to those with no history of COVID-19. A unit increase in the Fear-of-COVID score was associated with a 6% increase in the odds of non-adherence while a unit increase in the understanding or perception scores were associated with 95% and 24%, respectively, decrease in the odds of non-adherence (Table 4).

**Table 4:** Multivariable regression model for the predictors of non-adherence in the studied sample

Predictor variables	Odds Ratio	95% CI	*p*-value
Age (years)	0.97	0.94 – 1.00	0.071
Sex (female vs. male)	1.53	0.63 – 3.69	0.347
Marital status (married vs. others)	0.98	0.35 – 2.74	0.973
Number of comorbidities	1.46	0.73 – 2.93	0.288
History of COVID-19	5.36	1.79 – 16.1	0.003*
Fears-of-COVID-19 score	1.06	1.01 – 1.11	0.029*
Understanding score	0.05	0.01 – 0.29	0.001*
Perception score	0.76	0.62 – 0.92	0.006*
*Constant*	4.44		0.001*

Hosmer and Lemeshow Test: Χ^2^ = 3.68, *p*-value = 0.885. Overall classification = 86.3%, Nagelkerke R Square = 0.370

^*^Statistically significant at *p* < 0.05

*CI* Confidence Interval

## Discussion

Adherence to HD, prescribed medications, diet and fluid restrictions is essential to get the desirable outcomes and prevent complications in patients with ESRD [4, 18]. The purpose of this study was to evaluate the adherence of ESRD patients to HD and medical regimen during COVID-19 pandemic. Our study revealed that the pre-pandemic adherence of ESRD patients to HD and medical regimen was 88.3% (47.3% moderate, and 41.0% good adherence) which was higher than other several studies conducted before the pandemic and reported about 50% adherence rate [19–21]. However, our finding was consistent with an earlier study by Naalweh et al. [15] which showed that 40.5% and 55.5% of ESRD patients had moderate and good adherence behaviors, respectively.

The mean pre-pandemic scores of ESRD-AQ sub-domains in this study were consistent with those reported by Naalweh et al. [15] except for the mean HD attendance and medication adherence scores which were lower than Naalweh's study (268.3 vs. 296.36, and 160.2 vs 184.32, respectively) [15]. In contrast, our mean scores of the total adherence behavior and its sub-domains were all higher than those reported by Arad et al. [22] which reported a total adherence score of 513.6 compared to 919.8 in our study. Furthermore, non-adherence behaviors among ESRD patients showed wide variations in the literature ranging from 2% to 80.4% for dietary restrictions, 9.7%–75.3% for fluid restrictions, 15.4%–99% for medical prescriptions, and to HD attendance as 33.6%. [5, 23–26].

During the COVID-19 pandemic, our study findings showed that adherence of ESRD patients to HD and medical regimens were adversely affected by the pandemic, leading to significant rise of non-adherence rate from 11.7% to 19.5%. The attribute claimed for rising non-adherence among patients with ESRD was the psychological distress. A study by Lee et al. [27] showed that over 85% of the studied ESRD patients were anxious about having their dialysis treatments because of the increased risk of SARS-CoV-2 infection during transportation or during dialysis sessions. In our study, we assessed fears of COVID-19 among our patients using FCV-19S. Earlier studies using FCV-19S in the general population reported a mean score of 18.5 and suggested that a cutoff point of ≥ 17.5 considered as a predictive risk for development of anxiety, depression or other psychiatric symptoms [28, 29]. The mean score of FCV-19S among our ESRD patients was 18.8, which was consistent with the mean score reported in the general population. Moreover, our study findings were consistent with Bonenkamp et al. [29] in that ESRD patients on HD might cope better with the pandemic as they are less affected by measures of social distancing and have high resilience.

In our study, FCV-19S showed significant positive correlations with the pre-dialysis phosphorus and potassium. A study by Sousa et al. [30] showed that levels of serum albumin and dialysis adequacy decreased significantly, while phosphorus levels increased, during COVID-19 outbreak, and suggested that results were because of difficulties in dietary restrictions and diminished physical activity during the lockdown [30]. However, in our study, the FCV-19S score showed a significant negative correlation with the pre-dialysis PTH. This unanticipated finding could be explained by the significantly higher FCV-19S score among non-adherent patients who showed less pre-dialysis PTH, compared to adherent patients. Furthermore, the low pre-dialysis PTH was observed in many non-adherent patients in our sample who were either on calcimimetics or had had a partial parathyroidectomy (8 and 10 patients, respectively). Further studies with appropriate power are needed to investigate these observed correlations.

Several studies reported significant associations between adherence and patients' age, gender, residence, educational level, social support, vascular access, alcohol, and smoking [15, 31, 32]. Chan et al. and Allen et al. suggested that longer duration of HD was associated with good adherence as the patients understood the efficacy of dialysis and interacted well with complications [33, 34]. However, our study did not find any association between HD duration and adherence, which was consistent with an earlier study by Ibrahim et al. [19]. A study by Mukakarangwa et al. [35] identified several other barriers for adherence such as transportation, long distance, poverty and treatment-related complications. In our study, the main predictors of non-adherence to HD and medications were the history of COVID-19, understanding and perception scores, and the Fear-of-COVID score. Furthermore, our study agreed with Naalweh et al. [15] in that no correlation existed between the pre-dialysis serum phosphate and the adherence behavior score, emphasizing that ESRD-AQ did not correlate to most of laboratory parameters in ESRD patients.

Although most of the patients in this study were adherent to social distancing, home-isolation measures, using mask, gloves and handwashing, 6.3% of ESRD patients in this study had a PCR-confirmed diagnosis of SARS-CoV-2 infection. This finding was consistent with earlier studies [36–39]. A meta-analysis by Chen et al. [36] reported that the incidence of COVID-19 among ESRD patients on HD was 7.7%. Another study by Hsu et al. [37] reported that the prevalence of COVID-19 infection among HD patients in the United States was 5.5%. In China, studies reported that prevalence of COVID-19 among ESRD patients on HD was 4.3% [38]. Another study in London HD units reported 11% prevalence [39] . These findings support the argument that ESRD patients were highly vulnerable to have COVID-19 infection with an increased risk of morbidity and mortality.

This study had some limitations that should be considered while interpreting its findings. The relatively small sample size and recall bias of the self-report tool for adherence were the main limitations of this study.

In conclusion, adherence of ESRD patients to HD attendance, fluid and diet restrictions, and medications were adversely affected during the COVID-19 pandemic. High FCV-19S, and low understanding and perception scores were the main predictors of non-adherence. Patients on HD should be carefully monitored for non-adherence during the COVID-19 pandemic, to avoid the adverse consequences of poor adherence. Patient-tailored strategies should be implemented to improve adherence and mitigate the psychological impact of the pandemic on HD patients.

## Supplementary Information

**Additional file 1:** **Table S1.** Distribution of the adherence scores of the ESRD-AQ in the studied sample.

**Additional file 2:** **Table S2.** Distribution of studied patients according to their responses to Fears-of-COVID-19 statements (*N* = 205).

**Additional file 3:** **Table S3.** Correlations between the adherence scores, laboratory parameters, Intradialysis weight gain, and Fears-of-COVID-19 score in the studied sample (*N* =205).

## Data Availability

The datasets generated during and/or analyzed during the current study are available from the corresponding author on reasonable request. The data are not publicly available because of their containing information that could compromise the privacy of research participants.

## Abbreviations

COVID-19: Corona-virus disease 2019
DOPPS: Dialysis Outcomes and Practice Patterns Study
ESRD: End stage renal disease
ESRD-AQ: End-stage renal disease adherence questionnaire
FCV-19S: Fear of COVID-19 Scale
HD: Hemodialysis
RRT: Renal replacement therapy
IDW: Interdialytic body weight
