# Supplementary material for: Adherence to hemodialysis and medical regimens among patients with end-stage renal disease during COVID-19 pandemic: a cross-sectional study
Source: BMC Nephrol. 2022 Apr 9;23:138. doi: 10.1186/s12882-022-02756-0 (PMC8994066; doi:10.1186/s12882-022-02756-0)
Supplement: Supplementary file 1 — Additional file 1: Table S1. Distribution of the adherence scores of the ESRD-AQ in the studied sample. [file 12882_2022_2756_MOESM1_ESM.docx]

**S1. Distribution of the adherence scores of the ESRD-AQ in the studied sample**

| Item No. | ESRD-AQ subscales | Range | During Pandemic | Before Pandemic | *p*-value |
| --- | --- | --- | --- | --- | --- |
|  | **Adherence Behaviors**: |  | **Mean (SD)** | **Mean (SD)** |  |
| 14 | HD attendance | 100–300 | 257.6 (79.1) | 268.3 (66.0) | 0.005 * |
| 17 | HD shortening | 0–200 | 139.8 (64.5) | 144.9 (62.3) | 0.005 * |
| 18 | Duration of HD shortening *if shortened* | 0–100 | 67.2 (33.3) | 69.5 (32.7) | 0.013 * |
| 26 | Adherence to medication | 0–200 | 156.6 (48.4) | 160.2 (47.7) | 0.004 * |
| 31 | Adherence to fluid restriction | 0–200 | 143.4 (45.8) | 143.9 (45.9) | 0.157 |
| 46 | Adherence to diet restriction | 0–200 | 134.4 (51.5) | 134.6 (51.2) | 0.705 |
|  | *Total adherence score* | 0 - 1200 | 898.9 (192.2) | 919.8 (186.2) | 0.004 * |
|  | **Adherence Perceptions:** |  | **Mean (SD)** | **Mean (SD)** |  |
| 11 | Importance of HD attendance | 1 – 5 | 1.87 (0.85) | 1.46 (0.60) | 1.00 |
| 22 | Importance of medication adherence | 1 – 5 | 1.70 (0.78) | 1.71 (0.76) | 1.00 |
| 32 | Importance of fluid restrictions | 1 – 5 | 2.21 (0.96) | 2.20 (0.93) | 0.157 |
| 41 | Importance of diet restrictions | 1 – 5 | 2.14 (0.85) | 2.13 (0.75) | 0.317 |
|  | *Total perceptions score* | 4 – 20 | 16.4 (2.13) | 16.5 (2.11) | 0.083 |
|  | **Adherence Understanding (No/ Yes)** |  | **Yes, *n* (%)** | **Yes, *n* (%)** |  |
| 12 | Understand the importance of HD attendance | 0 –1 | 205 (100.0%) | 203 (99.0%) | 0.152 |
| 23 | Understand the importance of medication | 0 –1 | 202 (98.5%) | 201 (98.0%) | 0.700 |
| 33 | Understand the importance of fluid restrictions | 0 –1 | 203 (99.0%) | 195 (95.1%) | 0.020 * |
| 42 | Understand the importance of diet restrictions | 0 –1 | 194 (94.6%) | 193 (94.1%) | 0.827 |
|  | *Total understanding score,* mean (SD) | 0 - 4 | 3.92 (0.36) | 3.86 (0.48) | 0.022* |

SD: Slandered deviation, HD: hemodialysis, ESRD-AQ: End-Stage Renal Disease Adherence Questionnaire

^*^. Statistically significant at *p*<0.05
