# Supplementary material for: Adherence to hemodialysis and medical regimens among patients with end-stage renal disease during COVID-19 pandemic: a cross-sectional study
Source: BMC Nephrol. 2022 Apr 9;23:138. doi: 10.1186/s12882-022-02756-0 (PMC8994066; doi:10.1186/s12882-022-02756-0)
Supplement: Supplementary file 2 — Additional file 2: Table S2. Distribution of studied patients according to their responses to Fears-of-COVID-19 statements (N = 205). [file 12882_2022_2756_MOESM2_ESM.docx]

**S2. Distribution of studied patients according to their responses to Fears-of-COVID-19 statements (N = 205)**

| Fears of COVID-19 Scale | Agree | Neutral | Disagree |
| --- | --- | --- | --- |
| 1. I am most afraid of Corona  2. It makes me uncomfortable to think about Corona  3. My hands become clammy when I think about Corona  4. I am afraid of losing my life because of Corona  5. When I watch news and stories about Corona on social media, I become nervous or anxious.  6. I cannot sleep because I'm worrying about getting Corona.  7. My heart races or palpitates when I think about getting Corona. | 121 (59.0%)  118 (57.8%)  52 (25.4%)  77 (37.6%)  89 (43.4%)  54 (26.3%)  54 (26.3%) | 51 (24.9%)  28 (13.7%)  24 (11.7%)  35 (17.1%)  16 (7.8%)  12 (5.9%)  16 (7.8%) | 33 (16.1%)  58 (28.4%)  129 (62.9%)  93 (45.4%)  100 (48.8%)  139 (67.8%)  135 (65.9%) |
| Total Score, mean (SD), range = 18.8 (8.2), 7 - 35 |  |  |  |
