# Supplementary material for: Adherence to hemodialysis and medical regimens among patients with end-stage renal disease during COVID-19 pandemic: a cross-sectional study
Source: BMC Nephrol. 2022 Apr 9;23:138. doi: 10.1186/s12882-022-02756-0 (PMC8994066; doi:10.1186/s12882-022-02756-0)
Supplement: Supplementary file 3 — Additional file 3: Table S3. Correlations between the adherence scores, laboratory parameters, Intradialysis weight gain, and Fears-of-COVID-19 score in the studied sample (N =205). [file 12882_2022_2756_MOESM3_ESM.docx]

**S3. Correlations between the adherence scores, laboratory parameters, Intradialysis weight gain, and Fears-of-COVID-19 score in the studied sample (N =205)**

|  | Mean (SD) | 1 | 2 | 3 | 4 | 5 | 6 | 7 | 8 | 9 | 10 | 11 |
| --- | --- | --- | --- | --- | --- | --- | --- | --- | --- | --- | --- | --- |
| 1. Adherence behaviors | 919.8 (186.2) | 1.000 |  |  |  |  |  |  |  |  |  |  |
| 1. Adherence perceptions | 7.5 (2.13) | 0.220* |  |  |  |  |  |  |  |  |  |  |
| 1. Adherence understanding | 3.9 (0.36) | 0.247* | 0.019 |  |  |  |  |  |  |  |  |  |
| 1. Fears-of-COVID-19 score | 18.8 (8.15) | -0.150* | -0.103 | -0.192* |  |  |  |  |  |  |  |  |
| 1. Hemoglobin (g/dL) | 9.9 (1.80) | 0.101 | 0.032 | -0.221* | 0.082 |  |  |  |  |  |  |  |
| 1. Ferritin | 498.8 (309.41) | 0.215* | 0.008 | 0.041 | 0.104 | 0.136 |  |  |  |  |  |  |
| 1. Calcium | 8.5 (0.94) | 0.153 | 0.063 | 0.016 | -0.021 | 0.026 | 0.041 |  |  |  |  |  |
| 1. Albumin | 4.0 (0.46) | 0.060 | -0.010 | 0.035 | -0.060 | 0.325* | 0.029 | 0.141* |  |  |  |  |
| 1. PTH | 449.5 (404.46) | -0.154 | -0.015 | -0.189* | -0.184* | 0.093 | 0.007 | -0.201* | -0.065 |  |  |  |
| 1. Predialysis Phosphorus | 5.0 (1.37) | -0.112 | 0.076 | -0.060 | 0.158* | 0.122 | 0.070 | -0.164* | 0.092 | 0.287* |  |  |
| 1. Potassium | 5.0 (0.83) | -0.011 | -0.026 | -0.053 | 0.205* | -0.110 | 0.144* | -0.206* | -0.046 | -0.005 | 0.345* |  |
| 1. Intradialysis weight gain (IDW), kg | 2.8 (0.92) | -0.150 | 0.065 | 0.114 | 0.040 | -0.026 | -0.108 | -0.108 | -0.024 | 0.047 | 0.142* | 0.149* |

*. Statistically significant correlation coefficient (Spearman's rho), at *p*<0.05.

PTH: Parathyroid Hormone: SD: Standard Deviation
